# Erinaceus coronavirus persistence in hedgehogs (*Erinaceus europaeus)* in a non-invasive, *in vivo*, experimental setting

**DOI:** 10.3389/fvets.2023.1213990

**Published:** 2023-09-15

**Authors:** Luca De Sabato, Giovanni Ianiro, Francesca Manzia, Marina Monini, Barbara Chiappini, Ilaria Di Bartolo, Gabriele Vaccari

**Affiliations:** ^1^Department of Food Safety, Nutrition and Veterinary Public Health, Istituto Superiore di Sanità, Rome, Italy; ^2^Centre for the Recovery of Wild Fauna in Rome, LIPU, Rome, Italy

**Keywords:** *Merbecovirus*, *Erinaceus europaeus*, Erinaceus coronavirus (EriCoV), Coronavirus, European hedgehogs

## Abstract

In the last 20 years, new zoonotic CoV strains have emerged (SARS-CoV, MERS-CoV, and SARS-CoV-2), and new species have also been reported in animals. In Europe, the Erinaceus coronavirus (EriCoV) was recently described in *Erinaceus europaeus*. However, information on the prevalence and duration of viral shedding is unknown. In this study, feces samples were collected from 102 European hedgehogs hosted in the Center for the Recovery of Wild Fauna in Rome and analyzed for the presence of EriCoV RNA by Reverse Transcription-PCR. In total, 45 animals (44.1%) resulted positive for EriCoV at the first sampling and 63 (61.7%) animals were positive at the follow-up, which was performed from the 3rd to the 86th day. The duration of fecal virus shedding showed a mean duration of 22.8 days and lasted up to 62 days. Eighteen hedgehogs showed intermittent viral shedding. Phylogenetic analysis showed a correlation with EriCoV strains reported in Germany, the United Kingdom, and northern Italy. None of the EriCoV sequences showed the CD200 ortholog insertion, previously observed in strains isolated in animals from northern Italy. Interestingly, all but one animal revealed the presence in their feces of the same EriCoV sequences, analyzing the short genomic region at 3' spike gene and 5' ORF3a 500bp fragment (100% nt.id.) in both first and follow-up samples. This result suggests that animals were infected with the same strain during their stay at the center. Our results confirm that EriCoV can persist in hedgehogs for a long period, underlining that hedgehogs are an important commensal reservoir for *Merbecovirus*. A long duration of viral shedding increases the likelihood that the virus will spread in the environment.

## 1. Introduction

In the last 20 years, two novel coronavirus species of the genus *Betacoronavirus* have emerged worldwide, posing serious public health threats and causing severe respiratory diseases in humans. One species, classified as *Sarbecovirus* subgenus, the *severe acute respiratory syndrome-related coronavirus* (SARS-CoV and SARS-CoV-2), has been responsible for severe acute respiratory syndrome causing thousands of cases, with the latter emerging in China in 2019 causing the recent pandemic with more than 640 million cases and more than 6 million deaths (https://covid19.who.int/). One species, within the *Merbecovirus* subgenus, the *Middle East respiratory syndrome-related coronavirus* (MERS-CoV), is still circulating in the Arabian Peninsula. After the first SARS-CoV epidemic in 2004, surveillance studies in wildlife intensified, and bat species were established as a natural reservoir of these three zoonotic Betacoronaviruses (1).

Coronaviruses (CoVs) are classified into four genera, *Alphacoronavirus, Betacoronavirus, Gammacoronavirus*, and *Deltacoronavirus*. The strains belonging to the *Betacoronavirus* genus are divided into five subgenera, namely, *Embecovirus, Sarbecovirus, Merbecovirus, Nobecovirus*, and *Hibecovirus* (1). The genus *Betacoronavirus* includes several bat-associated viruses, which are known to infect humans and are a major public health concern, with human CoV HKU1 and OC43 belonging to *Embecovirus*, SARS-CoV and SARS-CoV-2 belonging to *Sarbecovirus*, and MERS-CoV belonging to *Merbecovirus* (1).

In 2013, a new group (clade C) within the *Betacoronavirus* genus was discovered in the western European hedgehog (*Erinaceus europaeus*) in Germany (2). The Erinaceus coronavirus (EriCoV) was also described in France, Poland, Italy, and the United Kingdom in *Erinaceus europaeus* (3–6), and the HKU31 was reported in China in *Erinaceus amurensis* (7, 8).

In Europe, hedgehogs are widespread and are becoming synanthropic species. They live even more in urban areas. The new habitats occupied by hedgehogs increase the frequency of animal–human contact, exposing humans to potential zoonotic agents such as CoVs (9, 10). In addition, bats and hedgehogs, both hibernators and insectivores, share nocturnal habits posing the risk of cross-species transmission of viruses among animals.

The ability of CoVs to cross the species barrier relies on spike-protein plasticity. The genome of CoV changes due to a high mutation rate (10^−3^ to 10^−6^ mutations per site per year) (11); the acquisition of insertions and deletions, or recombination, allows the virus to adapt to new hosts posing a potential threat to humans. Analysis of the receptor-binding region in the S protein of EriCoV revealed that it has only 58.0% amino acid identity with that of MERS-CoV, which suggests a possible low level of adaptation to the human host (5).

To date, information about the persistence of viral infection in hedgehogs is unknown. This study aimed to investigate the presence of EriCoV in hedgehogs living in the ecosystems of the urban area of Rome and determine the persistence of infection in this animal host in a non-invasive, experimental *in vivo* setting.

## 2. Materials and methods

### 2.1. Sample collection

One hundred two injured hedgehogs (*Erinaceus europaeus*) hosted in the Center for the Recovery of Wild Fauna in Rome (LIPU), between February and June 2021 (*n* = 37) and between September 2021 and April 2022 (*n* = 65), were involved in this study. The hedgehogs were rescued from rural and urban areas in the Rome province of the Lazio region (central Italy). The animals were recovering due to the presence of injuries, debilitation, several traumas due to predation, fall from the nest, intoxication, or weaning on the ground. The housing facility hosted animals in one room. Cages were daily cleaned and kept separately, one for each animal, but in air contact with each other. Shortly after the arrival of animals in the recovery center, their fecal samples were collected from the floor of the cages where animals were housed. Fecal samples from 58 out of 102 animals were repeatedly sampled, approximately every 2 days until complete recovery or death.

During the recovery period, 42 animals were released into the natural environment after physical recovery, while 60 animals died during their permanence in the center.

No modification of standard procedures of the Center for the Recovery of Wild Fauna was adopted, with respect to animal welfare, to conduct the study.

During the recovery at the center, three hedgehogs were kept together for 1 week in a cage before final release in their natural habitat, in order to acclimate to the wild habitat. For individual recognition, they were spray painted on their spines with different colors. Before collecting their feces individually, animals were shortly moved, before their release, to another cage, and their feces samples were collected.

### 2.2. EriCoV detection

Total RNA was extracted using the QIAmp Viral Mini kit (Qiagen, Milan, Italy) from 10% (w/v) fecal suspensions in diethyl pyrocarbonate (DEPC)-treated water, starting from 200 μl with an elution volume of 100 μl. The RNA was stored at −80°C or immediately used. RNA was extracted from 513 fecal samples and tested for the presence of EriCoV using a previously described reverse transcription PCR (RT-PCR), designed on the EriCoV genome, and annealing on the spike and ORF3a genes, overlapping the region of the homolog CD200 insertion (5). Sixty RNA samples, either resulted positive (*n* = 30) or negative (*n* = 30) to the EriCoV specific PCR, were tested for the presence of other CoVs with an additional broad range pan-CoV semi-nested RT-PCR (12).

The DNA amplicons were sequenced using the same PCR primers used for the one-step RT-PCR by Eurofins Genomics (Milan, Italy). Sequences were submitted to the NCBI database (https://www.ncbi.nlm.nih.gov) under the following accession numbers: OQ627968–OQ628036.

### 2.3. Phylogenetic analysis

A maximum likelihood (ML) phylogenetic tree was built using 69 EriCoV sequences obtained in this study, 13 *Merbecovirus* reference sequences, 8 EriCoV strains from Italy, and 1 EriCoV strain from the United Kingdom. The ML tree was built using IQ-TREE version 2 with 1000 bootstrap replicates, using the substitution model suggested by the software after the model test analysis (13).

### 2.4. Statistical analysis

Univariate analyses were performed to assess factors associated with EriCoV positivity at the first sample in hedgehogs considering the age group (juvenile, sub-adult, and adult), year and months of sampling, seasons (summer/autumn 2021 vs. winter/spring 2022), and positivity at the last sample associated with dead status or animal release. After the chi-square test (univariate analysis), adjusted *p*-values were calculated to assess the age group associated with EriCoV positivity in hedgehogs. The adjusted *p*-values were compared with the Bonferroni-corrected significance level, and according to Bonferroni *post-hoc* tests, pairwise comparisons were implemented (“pairwise Nominal Independence” from the R package companion). For years, seasons of sampling, and the status of animals at the last collected sample (death or alive before release), the chi-square test was applied; for months, due to the small sampling size, the exact Fisher's test was applied. Tests, tables, and graphs were produced using the R software v. 4.1.2 (https://cran.r-project.org).

## 3. Results

Fecal samples from 102 animals (European hedgehogs) were analyzed for detection of EriCoV by RT-PCR. In total, 45 out of 102 animals (44.1%) were positive for the presence of the viral RNA at the first sampling, which occurred shortly after their admission to the center (Table 1). To test the sensitivity of the EriCoV-specific PCR used in this study, designed on the EriCoV genome, 30 positive and 30 negative RNA samples were also subjected to a broad range pan-CoV RT-PCR. Negative samples were all confirmed by the second test. Conversely, among the 30 EriCoV-positive RNA, only 17 were further confirmed positive using the broad-range pan-CoV assay, and after sequencing, the PCR products obtained were confirmed as EriCoVs.

**Table 1:** Univariate analyses to assess factors associated with EriCoV positivity in hedgehogs.

**Characteristics**	**Total in group**	Positive^*^(%)	***P***
**Age** ^**^	*n =* 82		
Juvenile	18	11 (61.1)	0.5
Sub-adult	12	8 (66.6)	0.7
Adult	52	28 (53.8)	1
Adjusted alpha			0.0167
**Year**	*n =* 102		
2021	80	38 (47.5)	1
2022	22	7 (32.0)	
**Months**			
	*n =* 12		
February 2021	8	4 (50.0)	1
February 2022	4	2 (50.0)	
	*n =* 7		
March 2021	5	1 (20.0)	1
March 2022	2	1 (50.0)	
	*n =* 12		
April 2021	5	1 (20.0)	1
April 2022	7	1 (14.0)	
**Seasons**	*n =* 102		
Summer 2021	24	7 (29.2)	0.33
Autumn 2021/Winter 2022	88	33 (37.5)	
**Characteristics**	**Total in group**	**Positive** ^§^ **(%)**	***P***
**Health status**	*n =* 102		
Dead	60	27 (26.5)	0.53
Released	42	22 (21.5)	

^*^Number of animals detected positive at the first sampling. ^**^Information was available for 82 animals. ^§^Number of animals detected positive at the last sampling.

Among the 102 hedgehogs, 58 were repeatedly sampled. This group was followed up for a minimum of 3 days and up to a maximum of 83 days, with a mean and median of 25.9 and 19.5 days, respectively. The mean of days between successive samplings was 2.6, the median was 2.5, the minimum was 1, and the maximum was 7.2.

Considering the results from samples obtained from the same animal during the follow-up, the prevalence of infected animals, with at least one EriCoV-positive fecal sample, reached 61.7% (63 out of 102), since 18 animals that were negative in the first sample, resulted positive in the follow-up. Out of 49 animals, 32 were followed up, resulting in EriCoV positive (49 out of 102, 48%) at the last sampling before release (*n* = 27) or death (*n* = 22) (Table 1).

**Figure 1:**
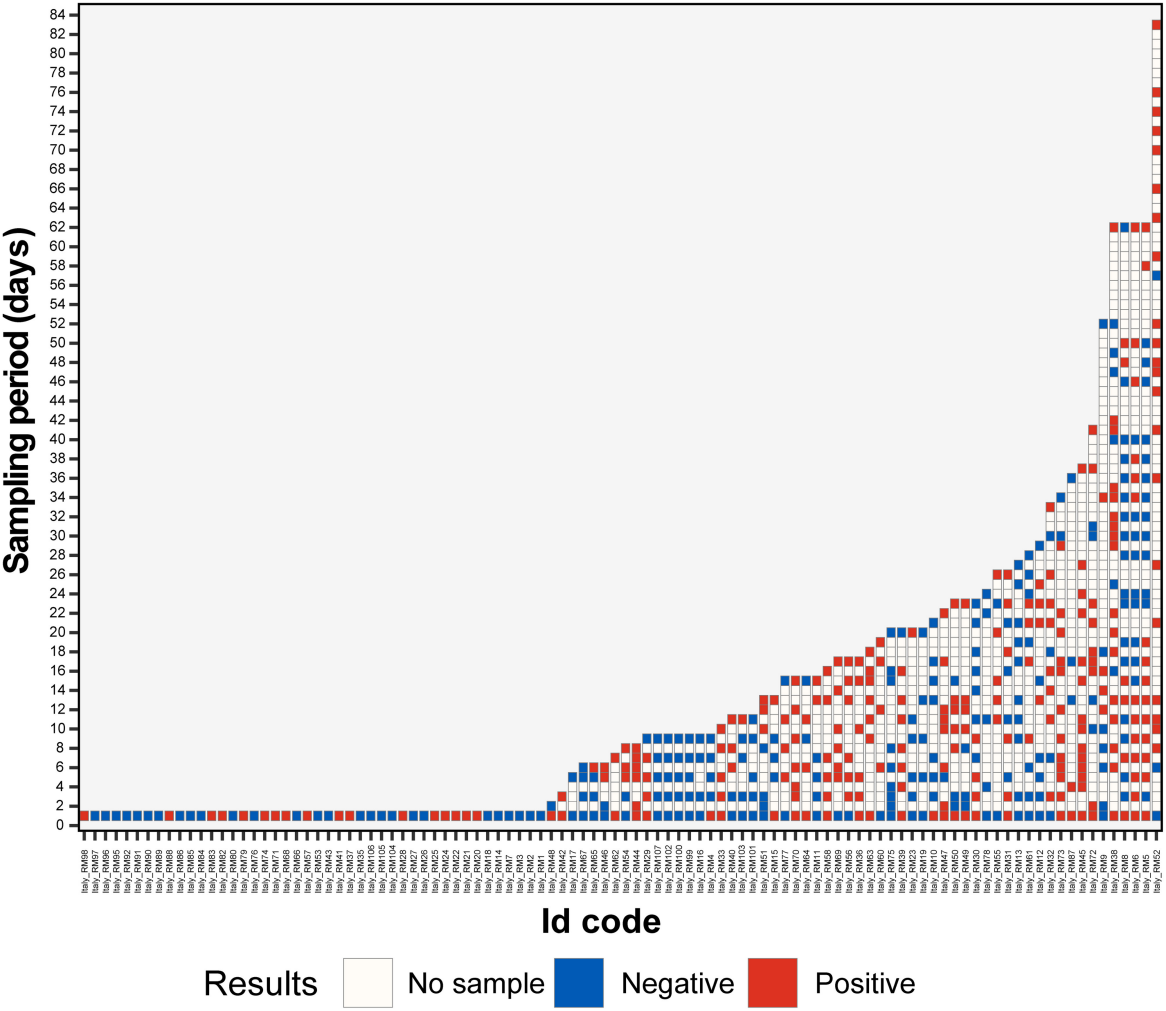
Results of hedgehog fecal samples screening with EriCoV RT-PCR. An alphanumeric code was assigned to each hedgehog, the animal codes are reported on the x-axis, and the length of sampling (in days) is reported on the y-axis. Positive (dark red) and negative (blue) PCR tests are color coded. The blank boxes correspond to the lack of samples.

The hedgehogs, which were repeatedly sampled, were grouped based on shedding duration (Figure 1). In order to evaluate shedding patterns, only animals with a long and continuous observation period were selected. Accordingly, animals followed up for more than 10 days with at least one sample, collected every 3 days, were used. Only 27 animals matched the established criteria (Supplementary Table 1), and the other animals were excluded from this analysis.

In total, 13 animals (13 out of 27) were followed up for 11–20 days, and 14 animals were followed up for more than 20 days (14 out of 27).

Interestingly, 7 out of 13 animals, among those followed up for 11–20 days, were positive for EriCoV in all fecal samples for a period of 15–17 days and were classified as long persistence shedders (LPS). Among the 14 animals followed up for more than 20 days, 7 had long viral shedding for a period of 17 to 37 days (2 animals for 29–37 days).

For the group of the 27 positive animals, we also determined the whole duration of shedding throughout the survey defined as the time (in days) between the first and the last positive sample. The maximum duration of the shedding period was 62 days, with mean and median of 22.8 and 16.0 days, respectively (range 3.0–62.0). Moreover, 55.5% (15 out of 27) of animals had a shedding period between 13 (2nd quantile) and 29 (4th quantile) days. The shedding period for 13 animals out of 27 corresponded to the rehabilitation period.

The univariate analysis of factors (age of animals, year, months, or seasons of sampling) and the occurrence of EriCoV RNA positivity in hedgehogs (*n* = 49) showed no significant correlation (detection rate *p* > 0.05; Table 1). Moreover, no significant correlation was detected between the occurrence of EriCoV positivity in the last sample analyzed and animal death (*n* = 27) or release (*n* = 22).

Considering the whole group (*n* = 58), 18 animals showed viral intermittent shedding (IS) being alternately positive or negative for EriCoV more times during the observational period. None of the animals followed up for <15 days showed intermittent shedding.

Among the 39 animals that resulted negative for the presence of EriCoV, 27 were tested only once after their arrival at the recovery center. The remaining 12, which resulted negative during the entire sampling collection period (from 2 to 14 samples) from 3 to 37 days, were defined as non-infected (NI). The NI group included also animals housed at the same time with hedgehogs of EriCoV-positive.

**Figure 2:**
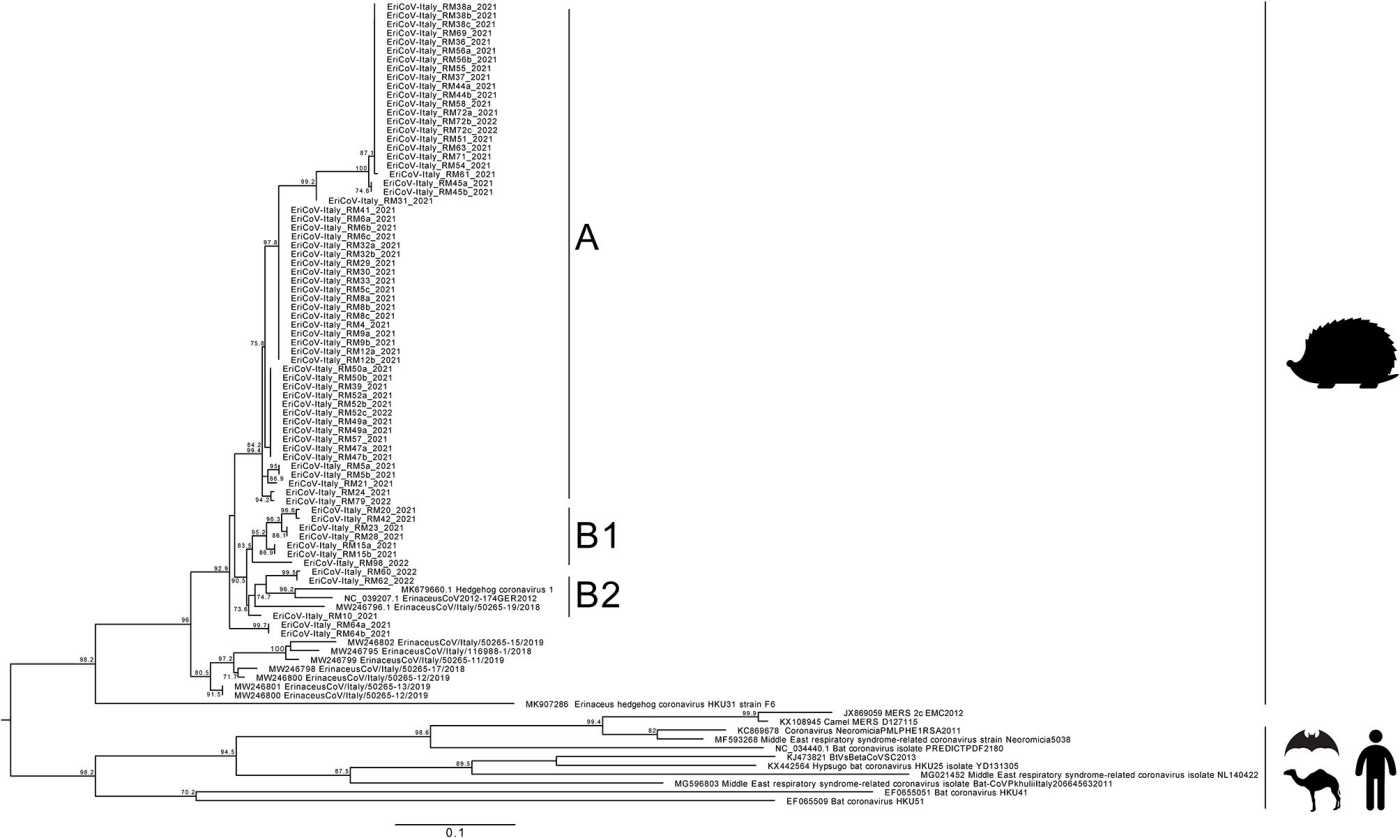
Maximum likelihood phylogenetic tree built with 99 sequences: 69 obtained in this study, 8 sequences from NCBI related to those from this study, and 18 *Merbecovirus* reference strains. The maximum likelihood tree was built using the TN (Tamura/Nei) model with invariant sites and gamma distribution based on 1,000 bootstrap replications, with bootstrap values of >70 indicated at their respective nodes.

Phylogenetic analysis was performed on nucleotide sequences obtained from strains detected in the feces of 48 animals, collected on the day of admittance in the recovery center. Sequences obtained showed 89.0–93.0% nucleotide identity (nt. Id.) with the EriCoV reference sequence Erinaceus/VMC/DEU/2012 isolate (Acc. Num.: NC_039207.1) and belonged to the same cluster (Figure 2). Interestingly, the insertion of CD200 orthologous, which was previously reported in ErinaceusCoV/Italy/50265-19/2018 sampled in northern Italy, was observed in none of the samples.

The maximum likelihood tree showed two clusters. The main cluster, named A, grouped 38 sequences from this study, displaying 93.0–100% nt.id. The second cluster was further separated into 2 subclusters: the former, named B1, included 6 sequences from this study (EriCoV-Italy_RM20, EriCoV-Italy_RM42, EriCoV-Italy_RM23, EriCoV-Italy_RM28, EriCoV-Italy_RM15, and EriCoV-Italy_RM98) sharing 92.0–100% nt.id. and the latter, named B2, included 3 sequences (EriCoV-Italy_RM60, EriCoV-Italy_RM62, and EriCoV-Italy_RM10) sharing 92.0–95.0% nt. Id. Strains included in subcluster B2 were correlated with the EriCoV strain from the United Kingdom (Hedgehog coronavirus 1, MK679660) (89.0% nt.id.) and with one strain from northern Italy (ErinaceusCoV/Italy/50265-19/2018, MW246796) (94.0–95.0% nt.id.). The sequence retrieved from one animal (EriCoV-Italy_RM64) was grouped out of clusters A, B1, and B2, showing nt. Id. Ranging between 91.0% and 95.0%.

No sequence clustering based either on the sampling period or on the age of animals was observed. Sequences obtained from animals either from the first or second year of sampling were interspersed and frequently identical to each other (e.g., sample EriCoV-Italy_RM23 and EriCoV-Italy_RM42).

In addition to sequences obtained from feces sampled on the first day in the center, a second positive sample from 17 animals was subjected to nucleotide sequencing. Among them, the second sample was sequenced from 2 LPS animals collected after 37 days of the first positive sample. For 15 animals (IS), the second sample sequenced was collected after a negative break ranging from 6 to 34 days. For five animals, collected between 49 and 82 days after the first sample, a third sample was also sequenced. All sequences obtained confirmed the presence of a single viral strain in each animal tested, presenting 100% nt. Id. And indicating the persistence in each animal of the same strain.

After the complete recovery and before their release into the natural environment, three hedgehogs were housed together in an open-air cage for 1 week to make them adapt to the natural conditions. Before their release, animal feces samples from every single animal were further tested and sequenced. One animal (EriCoV-Italy_RM8) tested negative when released, albeit a few weeks before the test, and its feces samples were EriCoV positive. One animal (EriCoV-Italy_RM6) was still positive for EriCoV, and sequences showed 100% nucleotide identity with previous samples. Conversely, one animal (EriCoV-Italy_RM5) resulted positive before its release, after a period of negativity of 4 weeks, showed an EriCoV sequence with a 98.0% nt. Id. To those obtained from previous fecal samples. It is noteworthy that the animal was infected by a different strain in the second sampling, which is identical (100% nt.id.) to the strain infecting animal EriCoV-Italy_RM6, cohoused before the release.

## 4. Discussion

In this study, the circulation of the EriCoVs was investigated in European hedgehogs in Rome, central Italy. Upon arrival at the recovery center, 44.1% of the animals tested positive for EriCoV in feces. The results confirmed the high prevalence of EriCoV in hedgehog species, similar to that observed in France 50.0% (3) and Germany 58.9% (2) but higher than reported in the United Kingdom 10.8% (6) and in Poland 25.0% (4). Differences between studies may depend on several factors, such as sample size, geographical area, climate, and the detection method used (4).

None of the EriCoV-positive animals showed signs or symptoms associated with an infectious disease during the inspection by veterinarians (data not shown). We cannot exclude the possibility that animals were also affected by other infectious diseases or that their health status was compromised by co-infection. Therefore, the high prevalence of EriCoV could be also linked to these factors.

However, previous studies did not observe any correlation between CoV positivity and the health of hedgehogs (2, 6, 15), as with Coronaviridae infections in bats and wild birds (16, 17). In addition, we did not find a correlation between either animal ages or sampling time (by year, months, or season sampling) and EriCoV positivity in animals, as previously reported (4, 6). Most of the animals (49) were still positive at the end of the survey before being released (27) or in the last sample before death (18). However, no statistical correlation was observed between the detection of EriCoV and these factors, excluding a correlation between these events.

In this study, we observed prolonged (mean 22.8 days) and intermittent viral shedding in the feces of hedgehogs, which may have been chronically infected, with a dynamic of infection similar to that reported for alphacoronaviruses in bats and cats (19, 20). The immune status of animals greatly influences the duration of infection and shedding. For the group of investigated animals, the long shedding period and the observed intermittence of shedding may depend on a reduced immune response, due to being injured and/or hosted outside their natural habitats (21).

Intermittent shedding has been described in cats infected by feline coronavirus (FcoV) (14, 22) and in humans affected by SARS-CoV-2 (18). In cats, viral shedding can last from 7 to 18 months (23); however, cats may undergo cycles of infection and shedding, recovery, and reinfection (24). A review study on viral shedding of SARS-CoV-2 reported a median duration of viral RNA detection from respiratory sources and fecal samples of 18.4 and 22.1 days, respectively, and intermittent shedding (18). A similar dynamic may apply to hedgehogs; some animals may be chronically infected, shedding EriCoV in feces over a long time and at a high viral load. Intermittent shedding (IS) may also occur in animals chronically infected for a long time, shedding the virus intermittently or with a different load. Indeed, we cannot rule out that the negative results depend on the viral load below the detection limit of the method used.

The present study has some limitations. The molecular method used did not allow viral load estimation which would have been useful to correlate IS with the viral load. Animals were injured, only feces samples were analyzed, and the observational period lasted until animals were released after rehabilitation or death. The health of animals could have influenced the prevalence of positive EriCoV observed compared with hedgehogs in their natural habitats or conditions. As previously described, EriCoV RNA concentration is higher in the aboral intestine than in other organs such as the lung or blood or urine (2). Subsequently, testing feces should not influence the result of the prevalence of EriCoV. The main limit is the sampling duration, which, if it had been followed up for longer, could allow for the observation of an even longer duration of shedding.

Nucleotide sequences obtained from different fecal samples collected in the follow-up of EriCoV-positive animals were identical (100% nt.id.), suggesting that the infection was caused by the same viral strain during the study. Another possibility is that the same animal was re-infected by the same strain. The 500 bp genomic region analyzed is highly variable, as observed by comparing Italian EriCoV genome sequences available online (NCBI accessed on June 2023), confirming that the 100% nt. id. observed could represent a good approximation of the whole genome relatedness. Future studies will be conducted to obtain the full genomes and confirm that a unique strain was involved.

All sequences identified in this study were closely related to the EriCoV sequences reported in the European species *Erinaceus europaeus* but significantly different from EriCoV strains detected in the other species *Erinaceus amurensis* in China (~85.0% nt.id.), suggesting a “species-dependent” clustering more than a geographical clustering.

Sequence analysis, although performed on a 500nt genome region, showed that different EriCoV strains circulate in the same province. However, since no clusters were observed based on the sampling period, seasons, or months, similar and identical strains co-existed at the same time in the hedgehog population over a long period (at least 2 years).

Phylogenetic analysis confirms the circulation of EriCoV strains in European hedgehogs in Italy, as previously reported (5, 15). Furthermore, the RT-PCR used for EriCoV detection was designed on genome sequences of other Italian EriCoV strains and overlapping regions of the CD200 orthologous insertion between genes coding the spike and ORF3a.

The insertion of the host CD200, a protein that regulates the immune response (5), into the EriCoV genome was recently described in EriCoV strains retrieved in a small population of hedgehogs in northern Italy. Conversely, none of the sequences obtained in this study showed this insertion, albeit the insertion into a different position of the genome could exist. Additional studies are necessary to verify the circulation in other areas of the country of animals infected by EriCoV strains with the CD200 insertion.

Seventeen animals tested negative for EriCoV at their arrival at the center but showed positivity in feces later, after 2–12 days of recovery. The hypothesis that animals may have become infected after sharing the same room with other EriCoV-positive animals already hosted in the same room could not be excluded. However, some animals that became EriCoV positive during their stay in the center showed EriCoV sequences different from those observed in the roommate animals. This result suggests that the negativity for EriCoV, when animals arrived at the Recovery Center, may depend on an effective negativity or a not detectable infection (a latent infection, an intermittent period, or an incubation period).

The housing system of animals in separated cages may prevent and reduce the EriCoV infection as suggested by eight animals negative for EriCoV during the whole permanence (from 3 to 37 days) in the center, despite the presence of positive animals in the same room. Otherwise, animals could have a phenotypic resistance to EriCoV. The susceptibility to coronavirus infection could be associated with factors related to the different immune responses of the host linked to its genome (25).

On the contrary, three hedgehogs kept together in the same cages before their release in their natural habitat, resulted in an intra-species transmission, as confirmed by the detection in one of the animals of different strains, before and after the co-housing period.

As described above, since animals may become infected inside the center, it is important to maintain good hygiene by frequent cleaning of the cages. Considering the high mutation rate of CoVs and their ability to recombine with other CoVs, leading to new viruses with potential for interspecies transmission, the separation between wild animals in the rehabilitation centers is extremely important. In addition, continuous surveillance of CoVs circulating in different wild animals is needed to understand the species circulation in wildlife and their possible inter- and intra-species transmission events.

## Data availability statement

The datasets presented in this study can be found in online repositories. The names of the repository/repositories and accession number(s) can be found at: https://www.ncbi.nlm.nih.gov/genbank/, OQ627968 - OQ628036.

## Author contributions

GV and ID: conceptualization. MM, GI, BC, and LD: methodology and investigation. LD: data analysis, writing, and editing. GV, ID, GI, and MM: review and supervision. GV: project administration and funding acquisition. All authors contributed to the article and approved the submitted version.
