# Supplementary material for: Erinaceus coronavirus persistence in hedgehogs (Erinaceus europaeus) in a non-invasive, in vivo, experimental setting
Source: Front Vet Sci. 2023 Sep 15;10:1213990. doi: 10.3389/fvets.2023.1213990 (PMC10545950; doi:10.3389/fvets.2023.1213990)
Supplement: Supplementary file 1 [file Table_1.pdf]

Group (sampling days)	ID	Sampling days	Shedding duration*	N. Collected Samples	N. Positive Samples	N. Negative Samples	Ratio Sampling days/N. Collected Samples	Max N. consecutive positive samples**	N° consecutive positive days§	Intermittent
10-20	Italy_RM40	11	6	5	3	2	2.20	3	6	
	Italy_RM101	11	0	5	0	5	2.20			
	Italy_RM103	11	0	5	1	4	2.20			
	Italy_RM15	13	13	5	2	3	2.60			Y
	Italy_RM51	13	4	7	3	4	1.85	3	4	
	Italy_RM11	15	3	6	2	4	2.50	2	3	
	Italy_RM70	15	15	7	7	0	2.10	7	15	
	Italy_RM77	15	7	5	3	2	3.00	3	7	
	Italy_RM64	15	11	5	3	2	3.00			Y
	Italy_RM58	16	16	6	6	0	2.66	6	16	
	Italy_RM36	17	17	6	6	0	2.80	6	17	
	Italy_RM56	17	15	7	1	6	2.40	7	16	
	Italy_RM69	17	13	7	1	6	2.40	6	13	
	Italy_RM63	18	18	7	7	0	2.57	7	18	
	Italy_RM39	20	16	8	7	1	2.50	7	16	
	Italy_RM75	20	0	9	0	9	2.20			
>21	Italy_RM10	21	0	8	1	7	2.60			
	Italy_RM47	22	22	9	8	1	2.40	6	17	Y
	Italy_RM30	23	9	11	4	7	2.10	3	5	Y
	Italy_RM49	23	23	8	5	3	2.80	4	14	Y
	Italy_RM50	23	23	9	6	3	2.50	4	6	Y
	Italy_RM31	26	26	10	6	4	2.60	2	4	Y
	Italy_RM13	27	0	10	0	10	2.70			
	Italy_RM61	28	15	11	4	7	2.50	2	3	Y
	Italy_RM12	29	13	10	4	6	2.90	3	5	Y
	Italy_RM32	33	31	12	7	5	2.75	3	6	Y
	Italy_RM73	34	29	14	12	2	2.40	12	29	
	Italy_RM45	37	37	14	14	0	2.60	14	37	
	Italy_RM8	62	50	23	17	6	2.70	6	7	Y
	Italy_RM5	62	62	24	11	13	2.60	8	15	Y
	Italy_RM6	62	62	22	13	9	2.80	7	13	Y
	Italy_RM38	62	62	23	17	6	2.70	6	13	Y

*number of days between the first and the last positive fecal sample
** number of consecutive positive samples
§number of days between the first and the last consecutive positive fecal samples
Y: intermittent shedding
